# Large malignant solitary fibrous tumour of the pleura and tumour doubling time: A case report and literature review

**DOI:** 10.1016/j.ijscr.2024.110404

**Published:** 2024-10-02

**Authors:** Yoshihito Iijima, Takaki Mizoguchi, Masahito Ishikawa, Shun Iwai, Nozomu Motono, Hidetaka Uramoto

**Affiliations:** Department of Thoracic Surgery, Kanazawa Medical University, Ishikawa, Japan

**Keywords:** Solitary fibrous tumour, Pleural tumour, Surgery, Tumour doubling time

## Abstract

**Introduction and clinical importance:**

Solitary fibrous tumours of the pleura (SFTPs) are rare, often benign, localized fibrous tumours. SFTPs are surgically excised after discovery. Herein, we report a rare case in which the tumour doubling time (TDT) was measured.

**Case presentation:**

A 71-year-old male patient with a history of cataract surgery presented with back pain and dyspnoea on exertion. Chest radiography revealed a large mass in the left thorax measuring 135 × 80-mm, after pleural effusion drainage. A thoracic tumour had been identified on a preoperative medical examination for cataract surgery 4 years previously; however, the patient did not opt for treatment. Chest radiography revealed a 43 × 22-mm mass. The TDT calculated using the Schwarz method, was 284 days. The tumour was resected and diagnosed as an SFTP. The postoperative course was uneventful, and the patient was discharged on postoperative day 9. No evidence of recurrence was observed one year and three months postoperatively.

**Clinical discussion:**

The malignant form of SFTPs remains unclear. The TDT of malignant SFPTs tends to be shorter than that of benign SFTPs in previous reports. However, in this case, despite the diagnosis of malignant SFT, the TDT was 284 days, which was longer than in previous reports.

**Conclusions:**

Compared with previous reports, there appeared to be no correlation between the risk of metastases and TDT. Few reports have calculated the TDT of SFPTs, and further accumulation of cases is desirable.

## Highlights

• Herein, we report a rare case of SFTPs in which the TDT was calculated.
• The malignant form of SFTPs still remains unclear.
• We observed no correlation between the risk of metastasis and TDT.
• Strict long-term follow-up is necessary in cases of malignant SFTPs.

## 1 Introduction

Solitary fibrous tumours (SFTs) are soft tissue tumours of mesenchymal origin arising from a wide range of anatomic sites. Approximately 68 % and 37 % of SFTs form in the thoracic cavity and the pleura, respectively [1,2]. Most intrathoracic SFTs arise from the visceral pleura [3], and SFTs of the pleura (SFTPs) represent <5 % of primary pleural neoplasms [4]. The incidence of SFTs is reportedly 2 % of all soft tissue tumours [5] and approximately 0.2 per 100,000 persons per year [5]. SFTPs, which have been reported in approximately 1500 cases previously, are rare, often benign, and localized [6]. SFTPs are slow-growing and relatively benign neoplasms, but up to 10 % of cases demonstrate aggressive behaviour [7]. A great percentage of SFTPs are asymptomatic and discovered incidentally [8]. Tumour doubling time (TDT) is often used as a prognostic factor for tumours and a parameter to predict tumour malignancy [9,10]. Since SFTPs are surgically excised shortly after their detection, few reports have calculated the TDT. Herein, we report a rare case of large malignant SFT in which the TDT was calculated, and the SFT was resected successfully. The study has been reported in line with the SCARE criteria [11].

## 2 Case presentation

**Fig. 1:**
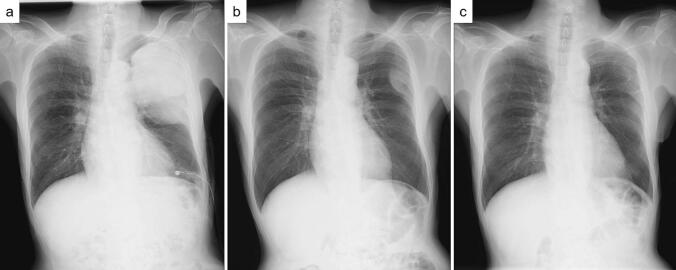
Chest radiography. (a) Preoperative chest radiograph showing a large mass in the left thorax measuring 135 × 80 mm after pleural effusion drainage. (b) A chest radiograph taken at the time of cataract surgery 4 years prior revealed a mass measuring 43 × 22 mm. (c) Follow up chest radiography at the time of 1 year and three months after surgery.

**Fig. 2:**
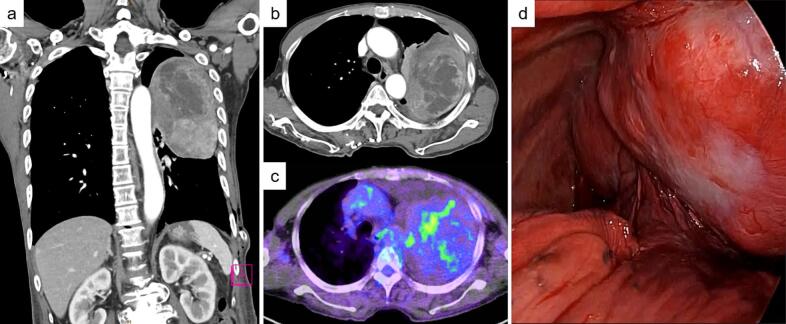
Calculation of tumour doubling time (TDT). t: time between the initial and second measurement. V_0_: tumour volume at the initial measurement; a_0_: maximum dimension of tumour at the initial measurement; b_0_: perpendicular dimension of tumour that crosses a_0_ at the midpoint; V_t_: tumour volume at the second measurement; a_t_: maximum dimension of tumour at the second measurement; and b_t_: perpendicular dimension of tumour that crosses a_t_ at the midpoint.

A 71-year-old male patient with a history of cataract surgery presented with back pain and dyspnoea on exertion. Chest radiography revealed a large mass in the left thorax measuring 135 × 80-mm after pleural effusion drainage (Fig. 1a). There were no symptoms of hypoglycaemia suggestive of Doege–Potter syndrome. No malignancy was detected on pleural effusion cytology. A thoracic tumour had been indicated during a preoperative medical examination for cataract surgery 4 years previously; however, the patient did not choose to be treated. Chest radiography revealed a 43 × 22-mm mass (Fig. 1b). The interval between the two examinations was 1529 days. The TDT calculated using the Schwarz method was 284 days [9,12]. Contrast-enhanced chest computed tomography (CT) revealed a tumour with heterogeneous contrast enhancement (Fig. 2a, b). Nuclear tracer accumulation in the tumour was visualised using 2-deoxy-2-(^18^F)-fluorodeoxyglucose positron emission tomography (Fig. 2c). Since the imaging findings did not rule out a malignant tumour, a decision was made to perform surgery for a definitive diagnosis.

Initially, we attempted to observe the left thoracic cavity using thoracoscopy. However, the tumour was large, making it difficult to expand the field of view (Fig. 2d). Consequently, an open thoracotomy was performed. The 5th intercostal muscle was resected along with the tumour. No obvious bone or chest wall invasion was observed cranially from the 5th rib, and the parietal extrapleural layer was detached. A feeding artery from the intercostal artery exists in the membranous structure of the 3rd intercostal space. The artery was cauterised and dissected using an ultrasonic coagulation device. The tumour grew, involving the lungs at three locations, and the involved lungs underwent combined partial resection. The duration of surgery was 172 min, and the amount of blood loss was approximately 220 g.

**Fig. 3:**
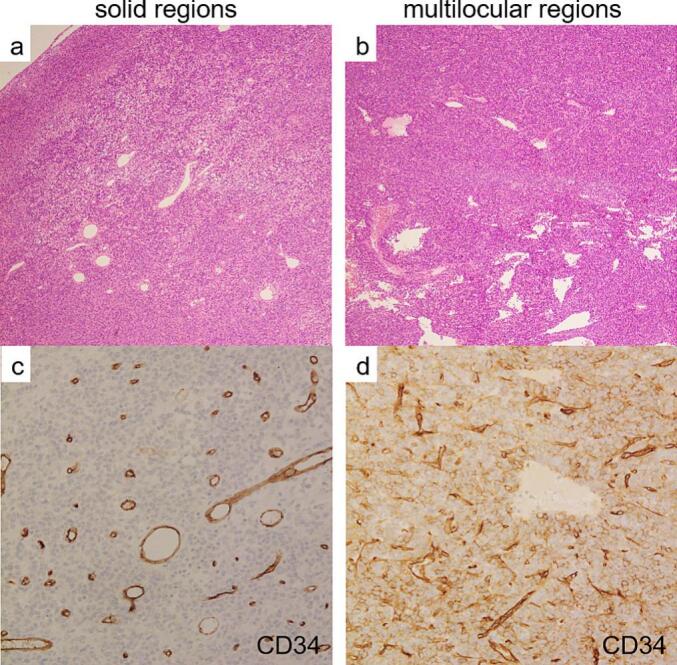
Contrast-enhanced computed tomography reveals a tumour with heterogeneous contrast enhancement; (a) Coronal view, (b) Horizontal view. (c) Nuclear tracer accumulation was visualised in the tumour on 2-deoxy-2-(18F)-fluorodeoxyglucose (FDG) positron emission tomography. (d) Intraoperative findings. The large tumour grew to involve the lungs in three places.

Macroscopically, the tumour was 14.0 × 11.5 × 4.5-cm with well-demarcated margins, a multilocular area of 9.5 × 9.0-cm, and a solid area surrounding it. Microscopy revealed that cells with spindle to ovoid-shaped nuclei, homogenously distributed fine chromatin, and inconspicuous nucleoli proliferated in the solid region, and no clear fascicle-like or alveolar-like patterns were observed (Fig. 3a). The background was rich in blood vessels and was partially staghorn-like. The density of the cells was diverse, and the interposition of collagen fibres was noticeable in some areas. The multilocular region showed a solid proliferation of small round cells with numerous microcystic structures (Fig. 3b). Mitotic cells were more abundant in the multilocular region, with seven per 10 high-power fields (HPF). Necrosis was observed in approximately 5 % of the tumour. All stumps tested negative. Immunohistological staining was positive for bcl-2 and STAT6 in both the solid and multilocular areas, and negative for AE1/3, CD31, CK5/6, D2-40, EMA, Factor VIII, and smooth muscle actin. CD99 and desmin were positive only in the solid regions, whereas CD34 was positive only in the multilocular regions (Fig. 3c, d). The Ki67 positive rate was approximately 20 % in the solid areas and 30 % in the multilocular areas. Therefore, SFTP was diagnosed, and complete resection was achieved. The chest drainage tube was removed on postoperative day (POD) 2. The postoperative course was good, and the patient was discharged on POD 9. No evidence of recurrence was observed one year and three months postoperatively (Fig. 1c).

## 3 Discussion

**Fig. 4:**
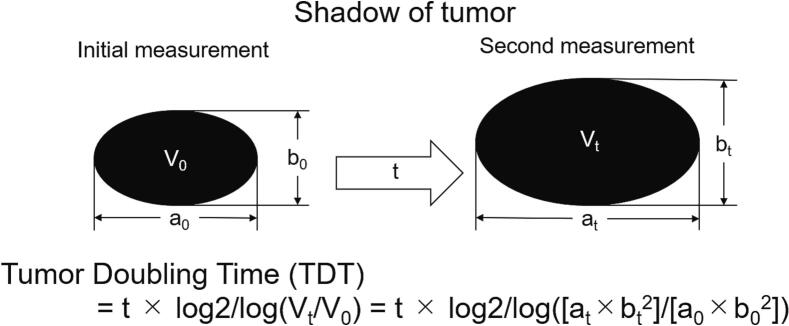
Microscopic findings. Hematoxylin-Eosin staining. (a) The cells with spindle to ovoid-shaped nuclei proliferate in the solid region, and no clear fascicle-like or alveolar-like pattern is observed. (b) The multilocular region has a solid proliferation of small round cells with numerous microcystic structures. Immunohistochemical staining. (c) CD34 was negative on solid regions. (d) CD34 was positive in multilocular regions.

Table 1 summarises the English and Japanese case reports of primary SFTPs for which TDTs could be calculated [4,[13], [14], [15], [16]]. TDT is often used as a prognostic factor for tumours and a parameter to predict tumour malignancy [9,10]. Moreover, TDT is useful for scheduling surgery and optimising the time interval from diagnosis to surgical resection. The Schwarz method is the most common method for calculating TDT [12]. (Fig. 4) In lung cancers, TDT has been reported as 161–180 days for adenocarcinomas, 80–100 days for squamous cell carcinomas, 29–69 days for small cell carcinomas, and 67–100 days for large cell carcinomas [9]. In previous reports, the TDT of SFTPs was calculated to be 41–241 days, except for one case that did not change significantly during the observation period (Case 6) [13]. In this case, the TDT was calculated as 284 days. This was considered relatively slow compared to previous reports. Excluding case 6 (benign) in which the TDT diverged indefinitely, the mean value for the three benign cases was 198.3 days, and the mean value for the three malignant cases, including one borderline malignant case, was 123.3 days.

**Table 1:** English and Japanese case reports of primary SFTP with calculated tumour doubling time.

Case	Age	Sex	Location	Size (cm)	Grade	Ki67	Mitosis (/HPF)	Necrosis	Interval (months)	Modality	TDT (days)	3-Variable risk model	4-Variable risk model	Reference
1	17	F	D	21	B	ND	rarely	ND	24	Xp	153	I	I	[13]
2	25	F	P	8	LGM	ND	scattered	ND	3	Xp	45	L/I^a^	L	[14]
3	56	F	V	9	B	ND	0	ND	48	Xp	201	L	L	[15]
4	67	F	P	11	B	ND	3	ND	37.7	CT	241	H	I/H^b^	[4]
5	70	F	P	7.1	M	ND	9–21	+	1	CT	41	I	I	[16]
6	54	M	V	3	B	ND	0	ND	36	Xp	∞	L	L	[15]
7	78	M	P	14	M	solid area: 20 % multilocular area: 30 %	7	5 %	52	Xp	284	H	I	Present case

HPF: high power field, TDT: tumour doubling time, F: female, M: male, P: parietal pleura, V: visceral pleura, D: diaphragm, B: benign, LGM: low grade malignancy, M malignant, ND: not described, Xp: X-ray photograph, CT: computed tomography, L: low, I: intermediate, H: high.

a Depending on the mitosis.

b Depending on the percentage of necrosis.

The majority of SFTs are benign, and approximately 5–45 % show aggressive clinical behaviour leading to local recurrence and/or metastatic disease; therefore, it is important to identify the malignant potential of SFTs to undertake informed clinical management [17]. The criteria for malignancy defined by England et al. include (i) abundant cellularity with crowding and overlapping of nuclei, (ii) high mitotic activity of >4 mitotic figures per 10 HPF, (iii) pleomorphism with cytonuclear atypia, (iv) large necrotic or haemorrhagic areas, (v) associated pleural effusion, and (vi) atypical location and inversion of adjacent structures [18]. This behaviour is often unpredictable and does not always correlate with histological findings [18]. By immunohistochemistry, CD34 is typically positive in most benign and malignant SFTPs. However, malignant SFTPs can be negative for CD34, which may be due to tumour differentiation. CD34 negativity may reflect poor prognoses [19]. The present case was diagnosed with a malignant SFTP because the mitosis was 7/HPF and necrosis and pleural effusion were observed. Moreover, CD34 expression was positive only in the multilocular regions and negative in the solid regions, possibly indicating that the solid regions of this tumour were high-grade malignant.

Complete *en bloc* surgical resection is the best treatment option for all benign and malignant SFTPs. The 5 and 10-year overall survival rates of malignant SFTPs are 81.1 % and 66.9 %, respectively [20]. Recurrence after complete resection occurs in 10–15 % of patients with SFTPs [21]. Demicco et al. reported that of 29 SFTPs in his case series, 12 cases experienced recurrence [22], including 3 local recurrences and 9 metastasis to the lung or pleura [22]. The 5-year overall survival rate of patients with complete resection was 87.1 %; in contrast, patients who underwent incomplete resection did not reach 5-year survival [19]. Two risk stratification models incorporating mitotic activity, necrosis, and tumour size have been validated; one model also includes patient age [[22], [23], [24]]. The risk of metastasis at 5 years of 3-viable and 4-viable risk models was 51 % and 73 % in the high-risk group, 7 % and 10 % in the intermediate-risk group, and 0 % and 0 % in the low-risk group, respectively [21]. These risk models for the prediction of metastatic risk in SFTPs were high risk for the 3-viable model and intermediate risk for the 4-viable model in the present case. The majority of SFTPs recur within the first 2 years after the initial resection [8]. Hence, after complete surgical resection in all patients, CT scans should be recommended to monitor recurrence every 6 months for the first 2 years and then annually [8]. Furthermore, all SFTPs are recommended to require long-term follow-up for >15 years owing to the possibility of late recurrence [8].

In conclusion, although, the TDT of malignant SFPTs tends to be shorter than that of benign SFTPs, there appeared to be no correlation between the risk of metastasis or its malignant potentials and TDT in this case. Few reports have calculated the TDT of SFPTs, and further accumulation of cases is desirable.

## Ethical approval

Ethics approval is not required for case reports in our institution as they are deemed not to be research.

## Funding

Not applicable.

## Author contribution

YI participated in the surgery, conceived and conducted the study, and performed the literature search. YI, MI, and HU participated in the surgery. TM and SI performed literature searches. NM and HU supervised the manuscript preparation and critically revised the manuscript. All authors have read and approved the final version of the manuscript.

## Guarantor

Yoshihito Iijima

## Consent

Written informed consent was obtained from the patient for publication and any accompanying images. A copy of the written consent is available for review by the Editor-in-Chief of this journal on request.

## Conflict of interest statement

All authors declare that they have no competing interests.

## Data Availability

All data generated or analyzed during this study are included in this published article.
